# *Mycobacterium pseudoshottsii* in Mediterranean Fish Farms: New Trouble for European Aquaculture?

**DOI:** 10.3390/pathogens9080610

**Published:** 2020-07-27

**Authors:** Davide Mugetti, Katia Varello, Andrea Gustinelli, Paolo Pastorino, Vasco Menconi, Daniela Florio, Maria Letizia Fioravanti, Elena Bozzetta, Simona Zoppi, Alessandro Dondo, Marino Prearo

**Affiliations:** 1 Istituto Zooprofilattico Sperimentale del Piemonte, Liguria e Valle d’Aosta, 10154 Torino, Italy; katia.varello@izsto.it (K.V.); paolo.pastorino@izsto.it (P.P.); vasco.menconi@izsto.it (V.M.); elena.bozzetta@izsto.it (E.B.); simona.zoppi@izsto.it (S.Z.); alessandro.dondo@izsto.it (A.D.); marino.prearo@izsto.it (M.P.); 2 Dipartimento di Scienze Mediche Veterinarie, Alma Mater Studiorum, 40064 Ozzano dell’Emilia (BO), Italy; andrea.gustinelli2@unibo.it (A.G.); daniela.florio@unibo.it (D.F.); marialeti.fioravanti@unibo.it (M.L.F.)

**Keywords:** atypical mycobacteria, slow-growing mycobacteria, *Mycobacterium marinum* complex, emerging diseases, European sea bass, gilthead sea bream, red drum, granulomas, gene sequencing

## Abstract

*Mycobacterium pseudoshottsii*, a slow-growing mycobacterium closely related to *M. marinum*, has been isolated only in wild fish in the United States and in Japanese fish farms to date. Here, we report cases of mortality in three farmed fish species (*Dicentrarchus labrax*, *Sparus aurata*, and *Sciaenops ocellatus*) caused by *M. pseudoshottsii* in Italy. Samples underwent necropsy, histology, and culture with pathogen identification based on PCR and sequencing of housekeeping genes (16S rRNA, *hsp65*, *rpoB*). Multifocal to coalescing granulomatous and necrotizing inflammation with acid-fast bacilli were observed in the parenchymatous organs, from which *M. pseudoshottsii* was isolated and identified. Phylogenetic analysis confirmed the results of gene sequencing and allowed subdivision of the isolates into three distinct groups. *M. pseudoshottsii* poses a potential threat for Mediterranean aquaculture. Its origin in the area under study needs to be clarified, as well as the threat to the farmed fish species.

## 1. Introduction

The scientific literature attributes a predominant role to *Mycobacterium marinum* in the onset of fish mycobacteriosis [1,2]. Its importance is well recognized; in fact, this mycobacterium can cause disease indifferently in freshwater and saltwater species, both farmed and wild species [3,4,5]. The use of biomolecular techniques in the diagnosis of mycobacteriosis has led to the discovery of many new etiological agents [6], including *M. shottsii* and *M. pseudoshottsii*, which are closely related to *M. marinum*. Both species were characterized following an epizootic event with mortality of wild striped bass (*Morone saxatilis*) in Chesapeake Bay, Maryland, USA [7,8,9,10].

The close similarity of *M. pseudoshottsii* to *M. marinum*, *M. ulcerans* (the causative agent of Buruli ulcer in humans), and *M. liflandii* (a frog pathogen) is based on analysis of the 16S rRNA gene and the presence of insertion sequence IS*2404* [1,11]. Since its isolation, *M. pseudoshottsii* has expanded its distribution area and range of fish species affected. It was first diagnosed in 2007 in white perch (*Morone americana*) in two rivers in Maryland (Rhode and Corsica rivers) and again in striped bass from Rockaway Beach, New York City [12]. Chesapeake Bay remained the most interesting area for studying *M. pseudoshottsii*. Its presence was found by real-time PCR in water, sediment, and in fishes (*Brevoortia tyrannus*, *Anchoa mitchilli*) preyed by striped bass, as well as in *M. saxatilis* [13].

These studies focused on fish in the natural environment; however, there is also a documented case of *M. pseudoshottsii* infection in farmed marine fish. Nakanaga et al. [14] described mortality events (1999–2008) resembling mycobacteriosis in farms in western Japan; positive fish belonged to five different species (*Seriola dumerili*, *S. quinqueradiata*, *S. radiata*, *Pseudocaranx dentex*, and *Epinephelus septemfasciatus*). Infected fish showed nonspecific clinical signs (skin ulceration, lethargy, anorexia, emaciation, abdominal distension with ascites), to which miliary nodules in liver and spleen were added at the necropsy. The authors also reported massive mortality in some batches of infected fish [14]. To our best knowledge, no other reports are available about fish mycobacteriosis owing to *M. pseudoshottsii*.

Mycobacterial infections are an important cause of concern for fish farms [4]. Cases have been documented in several European countries [15,16] and, specifically, in Italy [17,18,19,20]. To date, there have been no reports of infection due to *M. pseudoshottsii* in Europe. The present study reports on several mycobacteriosis events caused by *M. pseudoshottsii* in three marine fish farms in Italy.

## 2. Results

### 2.1. Gross Pathology

**Figure 1:**
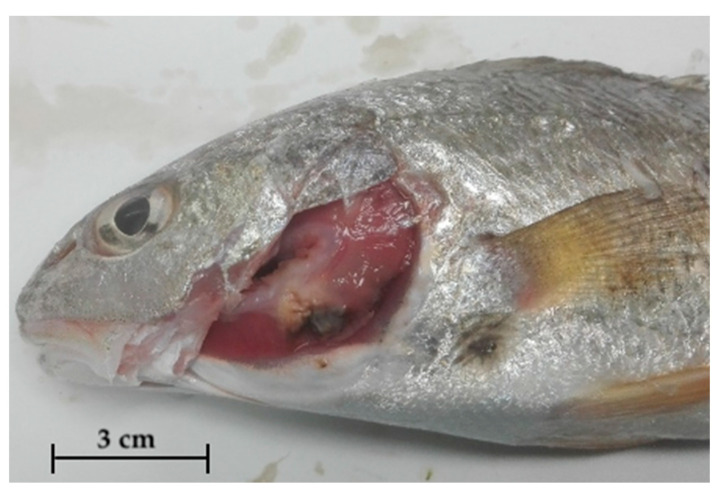
Gill necrosis in red drum (*S. ocellatus*).

**Figure 2:**
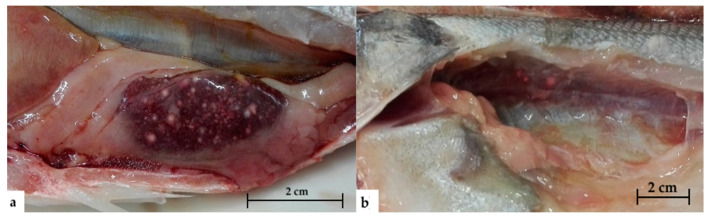
Nodules in enlarged spleen (**a**) and kidney (**b**) in European sea bass (*D. labrax*).

**Figure 3:**
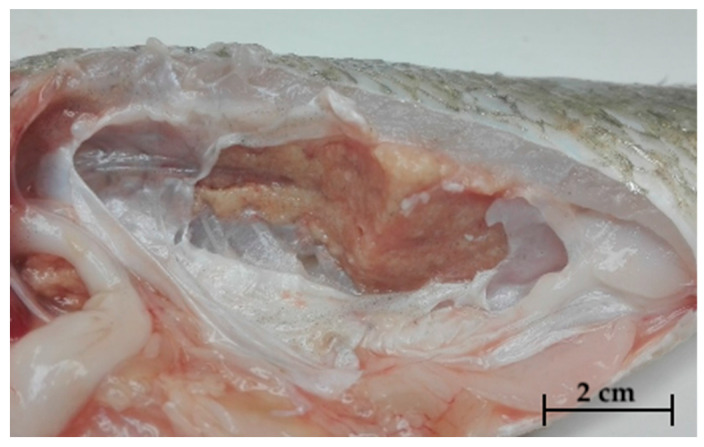
Discoloration and increased size owing to clustered multiple miliary nodules in the kidney of red drum (*S. ocellatus*).

An external examination showed skin erosions and ulcers, exophthalmos, and opercular erosions. Necrotic areas with abundant mucus on the gills were found in samples of red drum examined during 2019 (Figure 1). Internally, greyish-white nodules were found in liver (Figure 2A), spleen, and occasionally kidney (Figure 2B). With regard to red drum, it has been possible to observe such alterations in a more intense form, especially for renal parenchyma. Specifically, the kidney of these specimens showed discoloration and increased size owing to multiple grouped nodules (Figure 3).

### 2.2. Histopathological Findings

**Figure 4:**
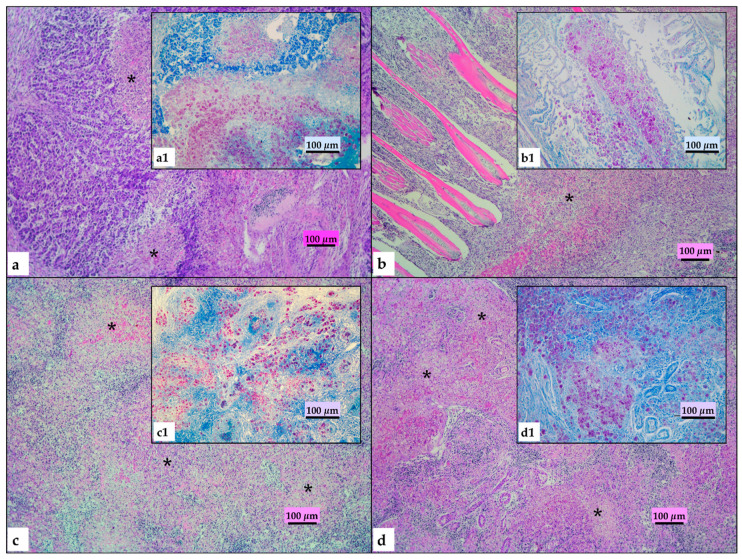
Histopathological features of granulomatous lesions in red drum. (**a**) Liver: multiple coalescing and necrotizing hepatitis with destruction of normal organ architecture (hematoxylin–eosin, HE); (**b**) gills: fusion of primary lamellae with severe granulomatous inflammation spread to the connective tissue of the gill arch; (**c**) spleen: multiple coalescing and necrotizing splenitis with destruction of normal organ architecture (HE); (**d**) kidney: multiple coalescing and necrotizing nephritis with destruction of normal organ architecture. Stars (*) highlight granulomatous and necrotizing lesions. (a1, b1, c1, d1) detail of large number of acid-fast bacilli in organs in bright red (Ziehl–Neelsen, ZN).

Histopathological examination disclosed lesions referable to mycobacteria infection in all organs of all examined animals. Most (70–80%) of the normal spleen and kidney tissue was replaced by multiple coalescing necrotizing granulomas. The granulomatous lesions were composed of epithelioid cells, foamy macrophages, lymphocytes, and plasma cells with large eosinophilic necrotic areas. Severe granulomatous inflammation was detected on the primary lamellae of the gills, extending to the base and infiltrating the connective tissue of the gill arch. All lesions displayed large amounts of acid-fast bacilli (Figure 4).

### 2.3. Mycobacteria Isolation and Species Identification

Culture grew colonies on Löwenstein–Jensen and Stonebrink medium from liver, spleen, and kidney samples from all three farms and fish species. The first colonies became visible after 6 weeks of incubation. Growth occurred at 28 °C, but not in the tubes incubated at 37 °C. Table 1 presents the bacteriological test results of positive and negative specimens.

All colonies tested for Kinyoun staining were alcohol-acid resistant, rod-shaped bacteria. PCR classified the colonies as strains of the genus *Mycobacterium*. Following sequencing and comparison with reference strains, all the isolates were identified as *M. pseudoshottsii*. All the sequences were highly similar to *M. pseudoshottsii* reference strain JCM 15466: nucleotide identities were 100% for both 16S rRNA and *rpoB*, and 99.76–100% for the *hsp65* gene. The sequences were deposited in GenBank under the following accession numbers: MT603289 (16S rRNA), MT603283, MT603284, MT603285, MT603286, MT603287, MT603288 (*hps65*), and MT603290 (*rpoB*).

### 2.4. Phylogenetic Analysis

**Figure 5:**
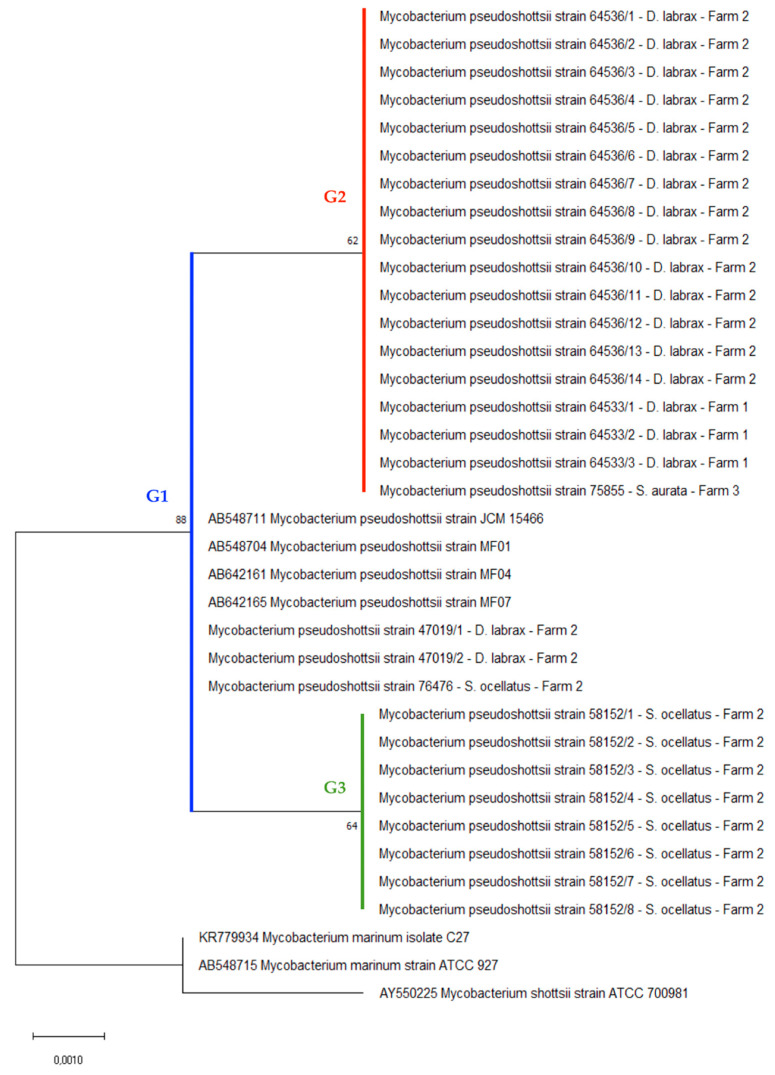
Phylogenetic tree constructed using partial *hps65* gene sequences (419 bp). The sequences were deposited in GenBank under MT603283 (*M. pseudoshottsii*, *D. labrax*, Farm 2, G1), MT603284 (*M. pseudoshottsii*, *D. labrax*, Farm 2, G2), MT603285 (*M. pseudoshottsii*, *D. labrax*, Farm 1, G2), MT603286 (*M. pseudoshottsii*, *S. aurata*, Farm 3, G2), MT603287 (*M. pseudoshottsii*, *S. ocellatus*, Farm 2, G1), and MT603288 (*M. pseudoshottsii*, *S. ocellatus*, Farm 2, G3).

Phylogenetic analysis performed with the *hsp65* partial gene sequences confirmed the results of Sanger sequencing. The phylogenetic tree showed three different genotypes of *M. pseudoshottsii*. One group (G1) includes the isolates from the USA, Japan, and several of the Italian strains. The other two groups (G2, G3) are composed entirely of Italian strains isolated from three different fish species. These groups form a cluster separate from the outgroups (Figure 5).

## 3. Discussion

Mycobacteriosis is a major problem for saltwater species farming. Although *M. marinum* is the main cause of clinical fish mycobacteriosis, new mycobacterial species responsible for mortality events have been continuously reported over the years. In the present study, the causative agent was *M. pseudoshottsii*, a species not yet reported for the Mediterranean basin according to our knowledge.

Three farms were sampled, two located on the South Adriatic coast and the other on the Tyrrhenian; *M. pseudoshottsii* was isolated in all three. Only one farm (Farm 2) was followed up for the two-year period, while the others provided fewer specimens. Further sampling will thus be necessary to understand whether the problem is only a sporadic finding. The same holds true for the fish species. European sea bass was the most numerous species analyzed and was the one with the greatest number of positive samples (19/70, p = 27.1%), confirming that mycobacteriosis is a constant problem for the breeding of this species [21,22,23]. Although the extent of mycobacteriosis appears to be less than in sea bass [22,24], *M. pseudoshottsii* was also isolated in sea bream. The entity of the findings in sea bream need to be clarified, as only one fish (1/10, p = 10%) tested positive. Finally, *M. pseudoshottsii* was also isolated in red drum (9/37, p = 24.3%). This finding is of particular interest, as only one case of mycobacteriosis in imported red drum from the Red Sea has been documented to date [25].

Another factor is the natural distribution of *S. ocellatus*; the Atlantic coast of North America [26] is the same area where most *M. pseudoshottsii* strains have been isolated [10,12]. Although there is no evidence for the importation of mycobacteria-infected red drum, the common geographical area suggests the need for further monitoring of this species and of imported batches in particular in view of pathogen control during fish translocation [27,28].

The pathological features of the fish we examined are shared by previously described cases of mycobacteriosis [4]. In red drum, the severe lesions were characterized by coalescing granulomatous and necrotizing inflammation in all organs that replaced normal parenchyma and were associated with numerous acid-fast bacilli. As the gill lesions were especially severe, it is conceivable that the gills provide a possible entry route for mycobacteria, as we previously suggested for European sea bass [29]. Experimental infection studies under controlled conditions are, however, needed to verify this hypothesis.

The first colonies took 6 weeks to grow on solid media, which complicates the diagnosis of *M. pseudoshottsii*. Nonetheless, the culture characteristics were fundamental for the diagnosis, especially for differentiating this species from *M. marinum*. While *M. marinum* can grow at both 28 °C and 37 °C, *M. pseudoshottsii* replicates with difficulty above 30 °C and is unable to grow at 37 °C [30]. Further testing is needed for identification, though this first screening may help in the diagnosis. In cases of suspected *M. pseudoshottsii*, incubation at temperatures below 28 °C can be a reliable tool to promote growth.

Gene sequencing was effective only with multiple genes owing to the close similarity between *M. pseudoshottsii* and other species. This multigenic approach has been suggested by previous studies [31], as there is no known single gene capable of discriminating against all *Mycobacterium* species. The 16S rRNA gene is the main target in bacterial species determination; however, the sequence of *M. pseudoshottsii* is identical to that of *M. marinum* [32]. The same is true for the *rpoB* gene, which lacks a suitable number of sequences for *M. pseudoshottsii* in public databases. In contrast, the *hsp65* gene has proven to be a more efficient means to discriminate between the two bacterial species, as reported in previous studies that showed that the *hsp65* gene exhibits greater variability than other genomic fragments [33,34].

Phylogenetic analysis of a portion of the *hsp65* gene confirmed the sequencing result and highlighted the presence of various *M. pseudoshottsii* genotypes. The three groups differed only in two nucleotides: sites 736 and 791 based on Shinnick’s work [35]. The isolates were grouped into G1 (C in 736 position, C in 791 position), G2 (C in 736 position, G in 791 position), and G3 (T in 736 position, C in 791 position). With this grouping, we were unable to observe the specificity of *M. pseudoshottsii* strains in relation to the farms and the fish species. Despite these nucleotide differences, the mutations are silent and the resulting amino acids remain leucine and threonine, respectively. Although the phylogenetic tree shows these differences between isolates, the nodes are not supported by bootstrap values. Nonetheless, the data highlight a genetic variability of *M. pseudoshottsii* strains not found in previous reports.

## 4. Conclusions

In this study, we isolated *M. pseudoshottsii* in farmed fish from the Mediterranean basin. It remains to be clarified whether *M. pseudoshottsii* was imported through infected fish or already present in the Mediterranean, as happened in the United States [36]. Focused attention is warranted as this pathogen belongs to a group of mycobacteria that produces mycolactone F, a powerful toxin [37] that causes necrosis and apoptosis and seems to be one of the main pathogenic mechanisms of these bacteria. Other mycolactone-producing mycobacteria are *M. marinum* and *M. ulcerans*, which are known human pathogens. Although the inability of *M. pseudoshottsii* to grow at 37 °C means that it is not a public health problem, marine fish farms may face a new threat of mycobacteriosis. Currently, there are no preventive and therapeutic measures effective against these bacteria. Fish elimination during massive disinfection is the only way to eradicate mycobacteria from a farm [4]. Indeed, mycobacteriosis can have a huge impact on fish production in intensive farming systems.

## 5. Materials and Methods

### 5.1. Fish Sampling

Three inshore farms were sampled: one located on the Tyrrhenian (Farm 1) and the other two on the Adriatic (Farm 2 and Farm 3) between May 2018 and July 2019. In 2018 (May to September), all three farms were sampled by the breeders’ request, while in 2019 (June to July), a thorough histopathological analysis of Farm 2 was performed. The tanks of the three farms are supplied by groundwater (average temperature 19–21 °C).

A total of 117 fish was analyzed. Farm 1: European sea bass (*Dicentrarchus labrax*) (n = 22, weight about 350 g); Farm 2: European sea bass (n = 48, weight about 400 g) and red drum (*Sciaenops ocellatus*) (n = 37, weight about 200 g); Farm 3: gilthead sea bream (*Sparus aurata*) (n = 10, weight about 2 kg) (Table 2). 

### 5.2. Anatomopathological Examination

Fish were killed by the farmers and sent to be refrigerated within 24 h to the Fish Diseases Laboratory of the Istituto Zooprofilattico Sperimentale del Piemonte, Liguria e Valle d’Aosta, Turin, where anatomopathological examination looked specifically for lesions attributable to mycobacteriosis. The liver, spleen, and kidney were collected for culture to detect non-tuberculous mycobacteria. In the red drum sampled in 2019, the organs were subjected to histology and culture; the gills underwent histology. Simultaneously, parasitological and bacteriological tests were conducted as described by Mugetti and coauthors to exclude pathogens other than mycobacteria [38].

### 5.3. Histopathological Examination

Tissues for histopathology were immediately fixed in 10% neutral-buffered formalin to avoid autolysis artefacts and processed by standard paraffin wax techniques. Samples were cut in 4 ±2 μm sections and stained with hematoxylin–eosin (HE) and Ziehl–Neelsen (ZN) histochemical acid-fast stain. Sections were observed microscopically at increasing magnifications (10x, 20x, 40x) on a Zeiss Axio Scope.A1 microscope (Zeiss, Jena, Germany). Mycobacterial lesions were evaluated and classified as described by Gauthier et al. [39] and the ZN stain was considered positive for the presence of bright red staining rods.

### 5.4. Culture Exams

The organs were mechanically homogenized with a stomacher in physiological solution. The homogenates were decontaminated with 1.5% cetylpyridinium chloride monohydrate (AppliChem, Darmstadt, Germany) solution for 30 min and then centrifuged for 20 min to obtain a pellet. A volume of 10 μl was inoculated using a sterile loop in mycobacterial selective media Löwenstein–Jensen (Microbiol, Uta-Cagliari, Italy) and Stonebrink (Microbiol, Italy). Two tubes were used for each medium, one incubated at 28 ± 1 °C and the other at 37 ± 1 °C for 2 months. All tubes were checked daily for bacterial growth. The colonies were tested for Kinyoun staining (ZN with cold-modified carbolfucsin). Positive colonies were subjected to biomolecular analysis for species determination.

### 5.5. DNA Extraction and PCR

Colonies were suspended in 200 μl of nuclease-free water (Sigma-Aldrich, St. Louis, MO, USA). DNA of the alcohol-acid resistant isolates was extracted by heating at 96 °C for 10 min and then freezing at −20 °C. After thawing and centrifugation to remove cell debris, the extracts were stored at −20 °C until analysis.

Three different PCR protocols were used to identify colonies; the targets were 16S rRNA [40], 65 kDa heat shock protein (*hsp65*) [41], and DNA-dependent RNA polymerase subunit β (*rpoB*) [42] (Table 3).

PCRs were performed on a 2720 Thermal Cycler (Applied Biosystems, Waltham, MA, USA) in a volume of 50 μl containing 1× TaKaRa Premix Ex Taq version 2.0 (Takara Bio Inc., Shiga, Japan) and 10 μM of each primer. *M. marinum* DSM 44,344 (ATCC 927) was used as PCR positive control and ultrapure water as negative control. Amplicons were revealed by electrophoresis on 2% agarose gel (Merck, Darmstadt, Germany), prepared using tris acetate-EDTA(ethylenediaminetetra-acetic acid) buffer 1× (Merck) and GelRed^®^ nucleic acid stain (Biotium, Fremont, CA, USA); the electrophoretic race lasted 50 min at 100 V and the molecular weight was determined by AmpliSize molecular ruler 50–2000 bp ladder (Bio-Rad, Segrate, Italy).

Amplicons were purified directly from gel using Extractme DNA Gel-Out kit (Blirt S.A., Gdańsk Poland) and sequenced in both senses by Sanger sequencing. Forward and reverse sequences were assembled with ClustalW [43]; the consensus sequence was compared with those present in the NCBI (National Center for Biotechnology Information) database using the nucleotide blast tool for species determination of the isolates.

### 5.6. Phylogenetic Analysis with hsp65 Gene Sequences

*hsp65* gene sequence portions (419 bp) were used to build a phylogenetic tree with MEGAX [44]. The tree was built predominantly with sequences of *M. pseudoshottsii* isolated in this study, in the United States (JCM 15466; accession number AB548711) [10] and Japan (accession numbers AB548704, AB642161, AB642165) [14]. A *M. marinum* refence strain (ATCC 927; accession number AB548715) [45] and an isolate of the same species from Italy (accession number KR779934) [20], plus *M. shottsii* (ATCC 700981; accession number AY550225) [8], were used as the outgroup. The statistical model was maximum likelihood analysis with a general time reversible (GTR) nucleotide substitution model; 1000 bootstrap replications were performed.

## Figures and Tables

**Table 1:** Bacteriological test results by farm and fish species (a): all positive fish were sampled in 2018; (b) 8 specimens of the 2018 sampling, 24 of the 2019 sampling; (c) one specimen of the 2018 sampling, 8 of the 2019 sampling; (d) 20 specimens of the 2018 sampling, 8 of the 2019 sampling.

		Fish Species					
		European Sea Bass (*D. labrax*)		Red Drum (*S. ocellatus*)		Gilthead Sea Bream (*S. aurata*)	
		Pos	Neg	Pos	Neg	Pos	Neg
**Farm**	Farm 1	3	19	-	-	-	-
	Farm 2	16 ^(a)^	32 ^(b)^	9 ^(c)^	28 ^(d)^		
	Farm 3	-	-	-	-	1	9

**Table 2:** Number of samples by farm and species. ^(a)^: 24 specimens sampled in 2018, 24 in 2019; ^(b)^: 21 in 2018, 16 in 2019.

		Fish Species			
		European Sea bass (*D. labrax*)	Red Drum (*S. ocellatus*)	Gilthead Sea Bream (*S. aurata*)	Total
**Farm**	Farm 1	22	-	-	22
	Farm 2	48 ^(a)^	37 ^(b)^	-	85
	Farm 3	-	-	10	10
	Total	70	37	10	117

**Table 3:** Primers for DNA amplification and sequencing.

Gene	Primer Forward	Primer Reverse	Length (bp)	Reference
16S rRNA	T_39_ (GCGAACGGGTGAGTAACACG)	T_I3_ (TGCACACAGGCCACAAGGGA)	924	[40]
*hsp65*	Tb11 (ACCAACGATGGTGTGTCCAT)	Tb12 (CTTGTCGAACCGCATACCCT)	441	[41]
*rpoB*	MycoF (GGCAAGGTCACCCCGAAGGG)	MycoR (AGCGGCTGCTGGGTGATCATC)	723	[42]
